# Structure of the gut microbiome following colonization with human feces determines colonic tumor burden

**DOI:** 10.1186/2049-2618-2-20

**Published:** 2014-06-17

**Authors:** Nielson T Baxter, Joseph P Zackular, Grace Y Chen, Patrick D Schloss

**Affiliations:** 1 Department of Microbiology and Immunology, University of Michigan, Ann Arbor, Michigan, USA; 2 Department of Internal Medicine, Division of Hematology and Oncology, University of Michigan, Ann Arbor, Michigan, USA

**Keywords:** Colorectal cancer, Community structure, Germ-free, Gut microbiome, Humanized mice, Microbiota

## Abstract

**Background:**

A growing body of evidence indicates that the gut microbiome plays a role in the development of colorectal cancer (CRC). Patients with CRC harbor gut microbiomes that are structurally distinct from those of healthy individuals; however, without the ability to track individuals during disease progression, it has not been possible to observe changes in the microbiome over the course of tumorigenesis. Mouse models have demonstrated that these changes can further promote colonic tumorigenesis. However, these models have relied upon mouse-adapted bacterial populations and so it remains unclear which human-adapted bacterial populations are responsible for modulating tumorigenesis.

**Results:**

We transplanted fecal microbiota from three CRC patients and three healthy individuals into germ-free mice, resulting in six structurally distinct microbial communities. Subjecting these mice to a chemically induced model of CRC resulted in different levels of tumorigenesis between mice. Differences in the number of tumors were strongly associated with the baseline microbiome structure in mice, but not with the cancer status of the human donors. Partitioning of baseline communities into enterotypes by Dirichlet multinomial mixture modeling resulted in three enterotypes that corresponded with tumor burden. The taxa most strongly positively correlated with increased tumor burden were members of the *Bacteroides*, *Parabacteroides*, *Alistipes*, and *Akkermansia*, all of which are Gram-negative. Members of the Gram-positive Clostridiales, including multiple members of *Clostridium* Group XIVa, were strongly negatively correlated with tumors. Analysis of the inferred metagenome of each community revealed a negative correlation between tumor count and the potential for butyrate production, and a positive correlation between tumor count and the capacity for host glycan degradation. Despite harboring distinct gut communities, all mice underwent conserved structural changes over the course of the model. The extent of these changes was also correlated with tumor incidence.

**Conclusion:**

Our results suggest that the initial structure of the microbiome determines susceptibility to colonic tumorigenesis. There appear to be opposing roles for certain Gram-negative (Bacteroidales and Verrucomicrobia) and Gram-positive (Clostridiales) bacteria in tumor susceptibility. Thus, the impact of community structure is potentially mediated by the balance between protective, butyrate-producing populations and inflammatory, mucin-degrading populations.

## Background

Colorectal cancer (CRC) is the second leading cause of cancer-related death in the United States each year [1]. Recent evidence suggests that the community of microbes inhabiting the gastrointestinal tract plays an important role in the development and progression of CRC [2-4]. This community, termed the gut microbiome, is known to influence cancer-related functions, including cell proliferation, angiogenesis, and apoptosis, and it is strongly linked to diet, obesity, and inflammation, which are known risk factors of CRC [5-9]. Using a mouse model of CRC, we have shown that structural changes to the microbiome occur during tumorigenesis and result in a gut microbiome with an increased tumorigenic capacity [10]. These findings demonstrate that the gut microbiome has a causal role in the development and progression of CRC.

Several survey-based studies have shown that CRC patients harbor microbial communities that are structurally distinct from those of healthy individuals [11-15]. However, there has been no consensus among these studies as to which bacterial populations are important. In mouse models, several gut commensals have been shown to promote tumorigenesis in the colon. Both enterotoxigenic *Bacteroides fragilis* (ETBF) and strains of *Escherichia coli* that carry the *pks* pathogenicity island can promote tumorigenesis by the production of toxins [3,4]. *Fusobacterium nucleatum* has also been shown to potentiate tumorigenesis in mouse models and cell culture experiments by stimulating inflammation via myeloid cell recruitment or activation of β-catenin signaling [2,16]. *Fusobacterium* was also found to be enriched in a subset of human colon adenomas [15]. Although there is increasing evidence that *Fusobacterium* is involved in CRC cases, it was detected in less than half of adenomas, which suggests that other bacterial populations are capable of potentiating tumorigenesis [2]. In fact, it may be that CRC is a polymicrobial disease requiring combinations of these or other populations to influence tumorigenesis.

While individual bacterial species have been associated with some human CRC cases, in other cases the capacity of the microbiome to modulate tumorigenesis could be determined by the structure of the community as a whole rather than the presence or absence of individual populations [4,17]. The potentially polymicrobial influence of the gut microbiome on this disease necessitates the disentangling of the complex interactions between bacterial populations in the gut. Understanding these interactions requires investigation of the relationship between the microbiome and tumorigenesis under a diverse set of community structures. Unfortunately, mechanistic studies typically rely on experiments with conventionally reared inbred mice living in homogenous, controlled environments, leading to relatively little variation in microbiome structure between individual animals. Although, experiments in conventional mice are useful for understanding the mechanisms by which the microbiome modulates tumorigenesis, they are limited by investigating only those bacterial strains found in laboratory mice, many of which are absent in human beings. It is reasonable to expect that incorporating human-associated microbial populations into these experiments would increase the ability to translate results to human beings.

To investigate the role of microbiome structure in tumorigenesis, we combined the advantages of the high interpersonal variation among human beings and the convenience of a mouse model. We inoculated germ-free mice with microbiota from human subjects harboring distinct microbiomes. This technique enabled us to test the effect of different baseline microbiome communities with variation beyond what is seen in conventionally reared mice. The transfer of human microbiota to germ-free mice, sometimes referred to as ‘humanization’, has been employed to study the microbiome in the context of several other diseases. In studies of diabetes, obesity, and malnutrition, colonization with human feces has been reported to recapitulate the phenotype of the human donors in the recipient mice [18-21]. Thus, in addition to searching for tumor-modulating community structures, we sought to determine whether this strategy could be used to recapitulate the tumor-promoting capacity of CRC patients’ microbiota in mice.

## Methods

### Mouse experiments

**Figure 1:**
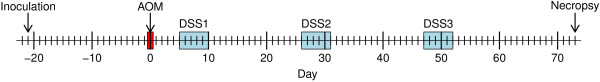
**Experimental design.** Germ-free mice were inoculated by oral gavage with one of six human inocula. Twenty-one days later (day 0), they received a single intraperitoneal injection of AOM (10 mg/kg). Mice were subsequently administered three five-day rounds of 2% DSS in the drinking water, with 16 days of rest in between. Mice were euthanized 73 days after the AOM injection for enumeration of colonic tumors. The inocula and samples collected on day 0 and day 73 were used for 16S rRNA gene sequencing. AOM, azoxymethane; DSS, dextran sulfate sodium.

Fecal samples from three healthy individuals and three patients found to harbor carcinomas were obtained through the Early Detection Research Network (Additional file 1: Table S1). Diagnoses were determined based on colonoscopy and histology. All six samples were PCR-negative for the ETBF toxin and the *E. coli pks* island [4,22]. Collection of the human feces used in this study was approved by the University of Michigan Institutional Review Board. All enrollees granted consent to participate in the study. Inocula were prepared by mixing 200 mg of each sample in 5 ml of PBS. Age-matched (6 to 10 weeks), male, germ-free C57BL/6 mice were inoculated by oral gavage with 100 μl of inoculum (*n* = 10 for groups H1 and C1, *n* = 5 for others). Mice were housed five mice per cage. Three weeks after inoculation, mice received a single intraperitoneal injection of azoxymethane (AOM; 10 mg/kg of body weight). Five days later, mice were subjected to the first of three five-day rounds of 2% dextran sulfate sodium (DSS) administered *ad libitum* in the drinking water (Figure 1). Sixteen days of recovery separated each round of DSS. Three weeks after the third and final round of DSS, mice were euthanized and colonic tumors were enumerated. With this model, mice consistently develop noninvasive adenomas with dysplastic changes [23,24]. Throughout the experiment, the mice were housed in germ-free isolators at the University of Michigan Germ-free Facility. This animal experiment was approved by the University Committee on Use and Care of Animals at the University of Michigan.

### DNA extraction and 16S rRNA gene sequencing

Mouse fecal samples were collected throughout the experiment and frozen at -20°C. Genomic DNA from samples collected on days 0 and 73 and the human inocula were isolated using the PowerSoil-htp 96 Well Soil DNA isolation kit (MO BIO, Carlsbad, CA, USA) using an epMotion 5075 automated pipetting system. The V4 region of the 16S rRNA gene was amplified using custom barcoded primers and sequenced as described previously using an Illumina MiSeq sequencer [25]. All fastq files and the MIMARKS spreadsheet are available online [26].

### Sequence curation and analysis

The 16S rRNA gene sequences were curated using the mothur software package, as described previously [25,27]. Briefly, paired end reads were assembled into contigs and aligned to the SILVA 16S rRNA sequence database [28]. Sequences that failed to align or were flagged as possible chimeras by UCHIME were removed [29]. Each sequence was classified using a naive Bayesian classifier trained against a 16S rRNA gene training set provided by the Ribosomal Database Project [30,31]. Finally, sequences were grouped based on their taxonomic classification or clustered into operational taxonomic units (OTUs) based on a 97% similarity cutoff. The number of sequences in each sample was rarefied to 3,306 sequences per sample, to minimize the effects of uneven sampling. Parallel sequencing and processing of a mock community indicated that the error rate of the curated sequences was 0.085%.

The dissimilarity in community structure between samples was calculated using the Θ_YC_ metric [32]. The Θ_YC_ distances between samples were used for ordination analysis by nonmetric dimensional scaling (NMDS) in two dimensions. Ten iterations were performed and the resulting ordination that had the lowest stress was used for data visualization. Dirichlet multinomial mixture models were generated to group samples into enterotypes based on the abundance of bacterial genera in each sample [33]. To identify conserved changes that occurred over the course of the AOM/DSS model, the samples from each mouse on day 0, and the samples collected at the end of the model were grouped into ‘baseline’ and ‘endpoint’ categories, respectively. The R randomForest package was used to identify the OTUs that best distinguished between the two categories based on their importance for the classification model [34,35].

The Phylogenetic Investigation of Communities by Reconstruction of Unobserved States (PICRUSt) software package was used to infer the metagenomic content of each sample, based on the taxonomy and abundance of each OTU [36]. Although this method is limited by the number of available genomes, it has been shown to replicate metagenomes to a high degree of accuracy, especially for human-adapted bacterial communities. The weighted nearest sequenced taxon index (NSTI) for our samples was 0.056 ± 0.01. In general, NSTI values below 0.06 suggest that closely related reference genomes were available for the dataset [37]. From the inferred metagenomes, we identified KEGG orthologs that could be used as markers for butyrate production or host glycan degradation. Because either butyrate kinase or butyryl-CoA:acetate CoA-transferase is required for butyrate production in the gut, the KEGG orthologs chosen as markers for butyrate production were K00929 (butyrate kinase (EC:2.7.2.7)), K01034 (acetate CoA-transferase α subunit (EC:2.8.3.8)), K01035 (acetate CoA-transferase β subunit (EC:2.8.3.8)) [38]. To choose markers for glycan degradation, we found all of the KEGG orthologs annotated as sialidases, fucosidases, sulfatases, or members of the glycoside hydrolase family 18, as these classes of enzymes are necessary, and moderately specific for host glycan degradation [39,40]. Ten such KEGG orthologs were found in the metagenomes and used as markers: K01138 (uncharacterized sulfatase (EC:3.1.6.-)), K01130 (arylsufatase (EC:3.1.6.1)), K01135 (arylsufatase B (EC:3.1.6.12)), K01137 (*N*-acetylglucosamine-6-sulfatase (EC:3.1.6.14)), K01134 (arylsufatase A (EC:3.1.6.8)), K01186 (sialidase-1 (EC:3.2.1.18)), K01206 (α-L-fucosidase (EC:3.2.151)), K01183 (1,4-β-poly-*N*-acetylglucosaminidase (EC:3.2.1.14)), K01205 (α-*N*-acetylglucosaminidase (EC:3.2.1.50)), and K05970 (sialate *O*-acetylesterase (EC:3.1.1.53)). Finally, we calculated the Spearman correlation coefficients between tumor counts and these KEGG orthologs.

### Statistical analysis

Differences in tumor counts between Dirichlet multinomial mixture (DMM) partitions were examined using a Wilcoxon rank-sum test. To test whether there was a significant difference in tumor counts between groups that received healthy or cancer-associated inocula, we rank transformed the tumor counts to correct for heteroscedasticity and performed a nested analysis of variance (ANOVA). Differences in community structure were examined using analysis of molecular variance (AMOVA) in mothur [41].

## Results

### Colonization of germ-free mice with human microbiota

**Figure 2:**
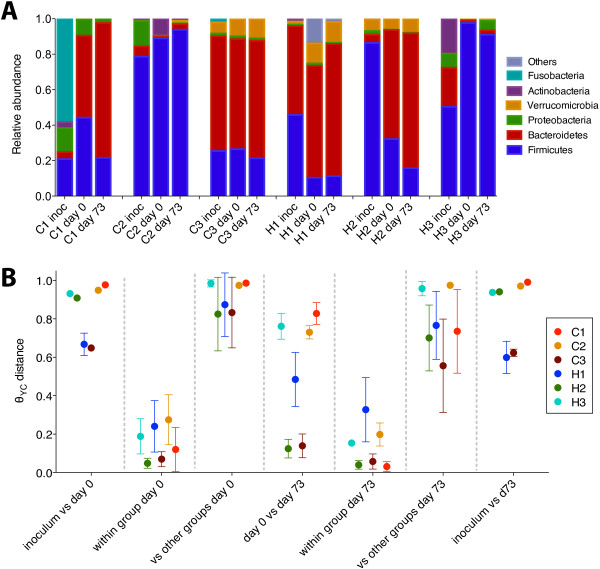
**Taxonomic composition and beta diversity across treatment groups and time. (A)** Phylum level relative abundance of the fecal microbiome of each group on days 0 and 73 and in its inoculum. **(B)** Average Θ_YC_ distances (±standard error in the mean) within and between groups at various time points; between each group and its inoculum, within each group at day 0, each group compared with others at day 0, between day 0 and day 73 for each group, each group compared with others at day 73, and between the inoculum and day 73.

We colonized germ-free mice with human feces from six individuals to determine whether different initial community structures would yield different numbers of tumors after going through the AOM/DSS model. This model was selected because the progression tumors in the AOM/DSS model closely resembles that of human CRC, including early mutations in APC or β-catenin signaling [42]. Furthermore, the model achieves colonic tumors and complete penetrance in the widely available C57BL/6 strain within 73 days. Three of the donors had healthy colons (H1, H2, H3) and three had colonic carcinomas (C1, C2, C3). Samples were chosen because they represented broad variation in community structure (Figure 2A). Following gavage and a 21-day colonization period, groups showed varying levels of similarity to their inocula based on phylum level relative abundances and the Θ_YC_ distances calculated from OTU abundances (Figure 2A,B). Low Θ_YC_ distances between mice within groups suggested that individual communities were consistent within each group, while large Θ_YC_ distances between groups suggested that each group harbored a gut microbiome that was structurally distinct from the others. Pairwise AMOVA between groups revealed that colonization with different inocula resulted in significantly different community structures (*P* < 0.01, Benjamini-Hochberg correction). These results suggest that although mice do not closely resemble their inoculum, all sets of mice developed stable, structurally distinct gut communities.

### Tumor incidence is linked to initial community structure

**Figure 3:**
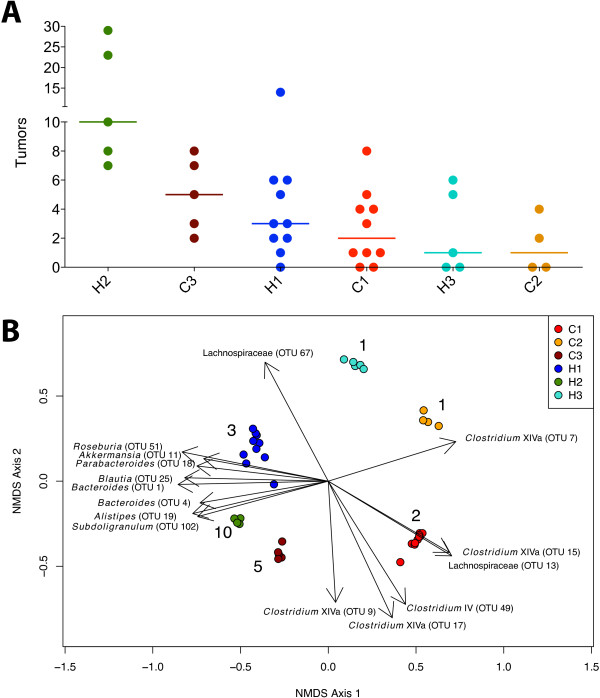
**Correlation of tumor incidence with initial gut community structure. (A)** Strip chart of tumor counts (with line at median) for each group. **(B)** NMDS plot based on Θ_YC_ distances between samples at day 0 with biplot of the 15 OTUs most strongly correlated with the NMDS axes (stress = 0.21). Median tumor counts for each group are adjacent to their corresponding dots. NMDS, nonmetric dimensional scaling; OTU, operational taxonomic unit.

Once colonized, mice were subjected to the AOM/DSS model of CRC. We observed significant variation in the number of tumors between mice (Figure 3A). These differences were associated with the inoculum they received, but not the cancer status of the human donor (nested ANOVA *P* < 0.0005). Thus, the phenotypes of the human subjects were not transferred to their mouse counterparts. Ordination of the communities revealed an association between the community structure of each group at the beginning of the AOM/DSS model and their median tumor counts (Figures 3B and Additional file 1: Figure S1). To test for cage effects, groups H1 and C1 were each inoculated into duplicate cages of five mice each (*n* = 10 per inoculum). There was no significant difference in baseline microbiome structure (*P* > 0.05, AMOVA) or tumor counts (*P* > 0.05, Wilcoxon test) between cages within each group.

To determine which OTUs were driving this trend, we generated a biplot using the NMDS axes generated from the Θ_YC_ distances between samples collected at the time of AOM injection (day 0; Figure 3B). Among the OTUs most strongly correlated with high tumor counts were two OTUs from the genus *Bacteroides* (OTUs 1 and 4). More detailed characterization of these OTUs indicated that OTU 1 was closely affiliated with *B. uniformis* and OTU 4 was affiliated with a mixture of *Bacteroides* species, including *B. fragilis*, *B. ovatus*, *B. xylanisolvens*, and *B. thetaiotaomicron*. Both of these OTUs were found in all six cohorts of mice and their initial abundances were positively correlated with tumor counts (*ρ* = 0.47 and 0.49, respectively; both *P* < 0.005; Spearman correlation). Interestingly, all samples were PCR-negative for the ETBF toxin gene, suggesting that OTU 4 was not ETBF. Other OTUs associated with high tumor counts were affiliated with the genera *Parabacteroides* (OTU 18) and *Alistipes* (OTU 19), as well as an OTU affiliated with the species *Akkermansia muciniphila* (OTU 11). In addition, several OTUs associated with *Clostridium* Group XIVa (OTUs 7, 9, 15, and 17), *Clostridium* Group IV (OTU 49), and unclassified members of the Lachnospiraceae (OTUs 67 and 13) were correlated with lower tumor counts. These results indicate that the relative abundance of specific OTUs in the starting community could be associated with tumor counts.

**Figure 4:**
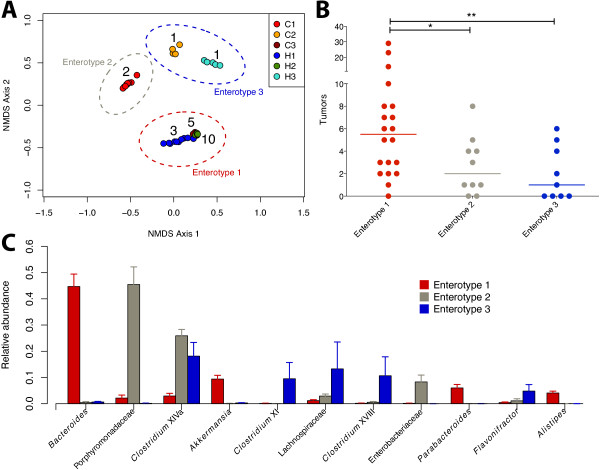
**Correlation of enterotypes with tumor incidence. (A)** NMDS plot based on genus level abundances with median tumor counts for each group (stress = 0.13). Samples are circled based on their DMM enterotype. **(B)** Tumor counts for the mice in each DMM enterotype (* *P* < 0.05, **P < 0.01, Wilcoxon rank-sum test). **(C)** Relative abundance of the genera with the largest differences between enterotypes. NMDS, nonmetric dimensional scaling.

To further test the association between the starting community structure and tumor incidence we clustered samples into community types using DMM models based on the abundance of bacterial genera found in the mice. This approach allowed us to quantify the association between the starting community structure and tumor burden in an unbiased manner. The DMM model with the highest likelihood partitioned the samples into three enterotypes (Figures 4A and Additional file 1: Figure S2). Enterotype 1 was composed exclusively of samples from the three treatment groups with the highest tumor counts (H2, C3, H1). Enterotype 2 was composed of samples from C1, which had the third lowest tumor count. Enterotype 3 was composed entirely of samples from the two groups with the lowest tumor counts (C2, H3). As a result, mice in enterotype 1 had significantly more tumors than the other two partitions (*P* < 0.05, Wilcoxon test; Figure 4B). Consistent with the OTU analysis, the DMM partition with the highest tumor counts was enriched for the genus *Bacteroides* (Figure 4C)*.* In addition, other genera within the order Bacteroidales (*Parabacteroides* and *Alistipes)*, as well as *Akkermansia*, were enriched in enterotype 1. An unclassified member of the Porphyromonodaceae, was enriched in enterotype 2, which had significantly fewer tumors than enterotype 1. Enterotype 3, which had the fewest tumors, was enriched for several genera within the order Clostridiales (*Clostridium* Group XIVa*, Clostridium* Group XI*, Clostridium* Group XVIII*, Flavonifractor*, and unclassified *Lachnospiraceae*). These data suggest a potentially tumorigenic role for certain members of Bacteroidales and a protective role for certain members of Clostridiales.

### Changes in the microbiome during the AOM/DSS model

To determine the extent to which the microbiomes of each group changed over the course of the AOM/DSS model, we calculated the Θ_YC_ distances between the communities in mice at the time of AOM injection and at the end of the experiment. Interestingly, the two groups with the highest tumor counts (H2, C3) changed very little over time (Θ_YC_ = 0.12 and 0.14), while the microbiomes of the three groups with the lowest tumor counts (C2, H3, C1) changed substantially (Θ_YC_ = 0.73, 0.76, 0.83) (Figure 2A). Thus, the closer the initial community of each group was to the tumor-associated endpoint community, the more tumors those mice developed.

**Figure 5:**
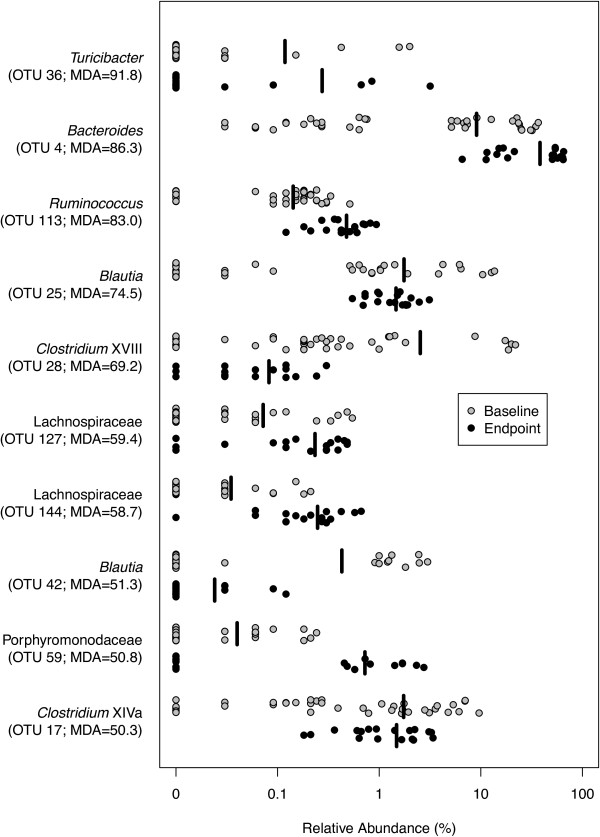
**Temporal changes in the microbiome are conserved between groups.** Strip chart showing the relative abundances of the ten OTUs with the highest importance for distinguishing between baseline (day 0) and endpoint (day 73) communities by Random Forest as measured by the MDA when the OTU was removed from the model. Each dot represents a single mouse. The black lines represent the mean relative abundance for all mice. MDA, mean decrease accuracy; OTU, operational taxonomic unit.

To identify which OTUs changed over time, we combined samples from all six treatment groups and used the Random Forest machine-learning algorithm to identify the OTUs that allowed us to differentiate between the samples from the beginning and end of the model, regardless of the inoculum. The resulting model was able to distinguish between the baseline and endpoint samples with 98.6% accuracy. The OTUs that provided the greatest mean decrease in accuracy when removed from the analysis were affiliated with *Turicibacter* (OTU 36), *Bacteroides* (OTU 4), Porphyromonadaceae (OTU 59), and several genera within the Clostridiales (OTUs 113, 25, 28, 127, 144, 42, and 17; Figure 5). Despite harboring drastically different community structures, all treatment groups underwent conserved changes in microbiome structure over the course of the model.

### Tumor incidence is linked to butyrate production and host glycan degradation

Our experiments suggested that Clostridiales, Bacteroidales, and *Akkermansia* played a role in modulating tumorigenesis. Members of the Clostridiales, especially *Clostridium* Group XIVa*,* are the predominate producers of intestinal butyrate, an important anti-inflammatory and anti-tumorigenic metabolite in the gut [38,43,44]. *Bacteroides* and *Akkermansia*, on the other hand, are known to break down host-derived glycans, especially mucin. Mucin degradation has been linked to intestinal inflammation and can facilitate colonization of intestinal pathogens [45-47]. To test whether the genomic potential for these metabolic activities is linked to tumor incidence, we used the PICRUSt software package to predict the metagenomic content for each sample at the time of AOM injection. Butyrate production in the gut requires either butyryl-CoA:acetate CoA-transferase or butyrate kinase [32]. KEGG orthologs (KOs) of the α and β subunits of butyryl-CoA:acetate CoA-transferase were negatively correlated with tumor incidence (*ρ* < -0.35, *P* < 0.05). Butyrate kinase had the same trend, but the correlation was not statistically significant (*ρ* = -0.30, *P* = 0.08). Next, we identified KOs for sialidases, fucosidases, sulfatases, and *N*-acetylglucosaminidases, which are indicative of host glycan degradation [39,40]. Of the ten such KOs found in our metagenomes, seven (two arylsulfatases, an uncharacterized sulfatase, α-L-fucosidase, sialate *O*-acetylesterase, α-*N*-acetylglucosaminidase, 1,4-β-poly-*N*-acetylglucosaminidase) were positively correlated with tumor count (*ρ* > 0.47, *P* <0.01). None of the three remaining KOs correlated with tumors. Together, these data suggest that the correlation between tumor incidence and the microbiome might be dependent on metabolic activity rather than bacterial phylogeny.

## Discussion

The results of this study demonstrate that the structure of the gut microbiome is important for determining susceptibility to inflammation-associated tumorigenesis. We observed strong correlations between the initial community structure of the gut microbiome and tumor multiplicity. This relationship is driven primarily by two distinct groups of bacteria. In general, we found that members of the Bacteroidales (*Bacteroides*, *Parabacteroides*, *Alistipes*, and Porphyromonodaceae) were associated with a higher rate of tumorigenesis, while members of the Clostridiales, especially *Clostridium* Group XIVa, were associated with a decreased rate of tumorigenesis. There were exceptions to this pattern, however, as a few OTUs associated with Clostridiales (OTUs associated with *Roseburia*, *Blautia*, and *Subdoligranulum*) were enriched in the groups with higher tumor counts (Figure 3B). However, these OTUs were less abundant (<0.7% mean abundance) than those Clostridiales that were negatively correlated with tumors (≈2% mean abundance). Therefore, the data generally support a model in which susceptibility to colonic tumorigenesis is determined by the balance between the abundance of members of Bacteroidales and Clostridiales. One limitation of this study is that we only assayed the fecal communities. While this was necessary for correlating baseline community structure with the numbers of tumors that developed, characterization of the mucosal microbiota could potentially yield additional associations with tumor burden. It is also important to note that, although we observed variation in the number of tumors within inoculum groups, we were unable to correlate these differences with any differences in their microbiomes.

Based on our predicted metagenomic analysis, the roles of Clostridiales and Bacteroidales could be dependent on specific metabolic activities. Members of *Clostridium* Group XIVa are the predominant producers of butyrate in the gut [38]. Given the anti-inflammatory and anti-tumorigenic properties of intestinal butyrate, its production by members of *Clostridium* Group XIVa could explain the association with lower susceptibility to colon tumorigenesis [43,44]. This hypothesis is supported by our predicted metagenomic data, which correlated the increased potential for butyrate production with decreased tumorigenesis. *Bacteroides* and *Akkermansia* were the two genera most strongly correlated with higher rates of tumorigenesis. Both are known mucin degraders, and several genes linked to mucin degradation were positively correlated with tumor incidence. Additionally, previous studies have linked mucin degradation by *Bacteroides* and *Akkermansia* with intestinal inflammation [45-47]. It is possible that an overabundance of these or other mucin degraders could undermine the integrity of the mucosal barrier, leading to increased inflammation. Such a mechanism could be an alternative to the ETBF-based model of tumorigenesis, as we were unable to detect the gene for the ETBF toxin in any of our samples. While we cannot exclude the possibility of a novel toxin in the *Bacteroides* populations in our experiment, the additional correlation with *Akkermansia muciniphila* supports a model in which inflammation is induced by mucin degradation. If further experiments confirm this model, blocking mucin degradation could be used as a therapeutic for preventing or slowing the progression of tumorigenesis.

In this study, we observed a relationship between tumor multiplicity and the extent to which the microbiome shifted over the course of the model. The gut community of mice with high tumor counts changed very little over the course of the model, while the microbiome of groups with low tumor counts changed drastically. Thus, the more similar the baseline community was to the endpoint community, the more tumors the host developed. We hypothesize that the microbiome of these mice was not significantly altered by the AOM/DSS model since it was already in a state of dysbiosis. Therefore, there was a greater exposure to a tumorigenic microbiome. Similarly, in a previous study, we colonized germ-free mice with the feces of conventional mice that had already gone through the model [10]. These mice developed more tumors than germ-free mice colonized with feces from normal mice. Thus, in addition to needing a dysbiotic community to exacerbate tumorigenesis, the length of exposure to that community is important to tumor formation.

In contrast with earlier studies where human feces were used to colonize germ-free mice, we were unable to recapitulate the structures of the human microbiota donors, as numerous members of the donor community failed to colonize the recipients and others colonized in different abundances. For example, one of the donor communities (C1) was dominated by *Fusobacterium* species (58% relative abundance). Another inoculum (C3), contained *F. nucleatum* at 2% relative abundance [2]. However, we did not recover any sequences from the phylum Fusobacteria in the recipient mice. We were also unable to culture it from the original human stool sample, suggesting that it might not have survived the freezing and thawing of the sample or was never alive in the stool. Colonizing germ-free mice with human feces and recovering a similar microbiome is probably an unreasonable expectation. The mouse host certainly selects for specific populations of bacteria based on its immune system and metabolic profile [48,49]. In addition, some bacteria will only colonize after other bacteria have suitably prepared the environment. In the oral cavity, *Fusobacterium* only colonizes after streptococcal populations have first attached to the tooth surface [50]. Although we did not fully recapitulate the community structure or phenotype of the human donors, colonizing mice with human fecal communities did serve as a useful tool for generating novel community structures to test the influence of specific bacterial populations on tumorigenesis. This strategy also allowed us to investigate the role of human microbiota, which should be more clinically relevant, while maintaining the tractability of a mouse model.

## Conclusions

In this study, we found that the process of colonizing germ-free mice with human fecal communities did not recapitulate the phenotype of the human donors in this particular mouse model of CRC. Nonetheless, our findings demonstrate the importance of the initial microbiome structure in determining the rate of tumorigenesis. Furthermore, we identified several bacterial populations correlated with tumor incidence in the context of six distinct gut communities. Multiple OTUs associated with the order Bacteroidales and the species *Akkermansia muciniphila* were correlated with exacerbated tumorigenesis, while several OTUs associated with *Clostridium* Group XIVa and other Clostridiales were correlated with protection. Based on inferred metagenomes of the baseline communities, we provided evidence that the positive correlations between *Akkermansia* and Bacteroidales and tumor incidence could be a result of their ability to degrade mucin, and the negative correlation between the Clostridiales and tumor incidence could be due to the production of butyrate. The results are consistent with a model in which susceptibility is determined by the balance between mucin degradation and short chain fatty acid production. More studies are needed to confirm these results and to test the mechanisms by which these or other bacterial populations influence colon tumorigenesis. A better understanding of microbiome structures with a propensity to promote or inhibit tumorigenesis could lead to the development of prebiotic or probiotic therapies to prevent or slow the development and progression of CRC.

## Abbreviations

AMOVA: analysis of molecular variance; ANOVA: analysis of variance; AOM: azoxymethane; CRC: colorectal cancer; DMM: Dirichlet multinomial mixture; DSS: dextran sulfate sodium; ETBF: enterotoxigenic *Bacteroides fragilis*; KO: KEGG ortholog; NMDS: nonmetric dimensional scaling; NSTI: nearest sequenced taxon index; OTU: operational taxonomic unit; PBS: phosphate-buffered saline; PCR: polymerase chain reaction; PICRUSt: Phylogenetic Investigation of Communities by Reconstruction of Unobserved States.

## Competing interests

The authors declare that they have no competing interests.

## Authors’ contributions

NTB and JPZ designed and carried out the study, performed the bioinformatic and statistical analysis, and wrote the manuscript. GYC and PDS helped design and write the manuscript. All authors read and approved the final manuscript.

## Supplementary Material

Additional file 1: Table S1 Metadata for the six inoculum donors. **Figure S1.** Temporal changes in community structure. NMDS ordination based the differences in OTU abundances between samples on day 0 and day 73. Distances were calculated with Θ_YC_. **Figure S2.** Samples remain in same enterotypes over the course of the model. NMDS ordination showing DMM enterotypes generated based on genus level abundances on day 73. Distances were calculated with Θ_YC_. Despite changes in OTU abundance over the course of the model, all mice clustered into the same enterotypes on day 73 as they did on day 0. Click here for file
