# Supplementary material for: Structure of the gut microbiome following colonization with human feces determines colonic tumor burden
Source: Microbiome. 2014 Jun 17;2:20. doi: 10.1186/2049-2618-2-20 (PMC4070349; doi:10.1186/2049-2618-2-20)
Supplement: Additional file 1: Table S1 — Metadata for the six inoculum donors. Figure S1. Temporal changes in community structure. NMDS ordination based the differences in OTU abundances between samples on day 0 and day 73. Distances were calculated with ΘYC. Figure S2. Samples remain in same enterotypes over the course of the model. NMDS ordination showing DMM enterotypes generated based on genus level abundances on day 73. Distances were calculated with ΘYC. Despite changes in OTU abundance over the course of the model, all mice clustered into the same enterotypes on day 73 as they did on day 0. [file 2049-2618-2-20-S1.docx]

**Table S1. Metadata for the six inoculum donors.**

**Figure S1. Temporal changes in community structure.** NMDS ordination based OTU abundances between samples on day 0 and day 73. Distances were calculated with Θ__.

**Figure S2. Samples remain in same enterotypes over the course of the model.** NMDS ordination showing DMM enterotypes generated based on genus level abundances on day 73. Distances were calculated with Θ__. Despite changes in OTU abundance over the course of the model, all mice cluster into the same enterotypes on day 73 as they did on day 0.
